# Regular Healthcare Provider Status Does Not Moderate Racial/Ethnic Differences in Human Papillomavirus (HPV) and HPV Vaccine Knowledge

**DOI:** 10.3390/vaccines9070802

**Published:** 2021-07-20

**Authors:** Trisha L. Amboree, Kalyani Sonawane, Ashish A. Deshmukh, Jane R. Montealegre

**Affiliations:** 1 Department of Epidemiology, The University of Texas Health Science Center School of Public Health, Houston, TX 77030, USA; Trisha.L.Amboree@uth.tmc.edu; 2 Department of Management, Policy and Community Health, The University of Texas Health Science Center School of Public Health, Houston, TX 77030, USA; kalyani.b.sonawane@uth.tmc.edu (K.S.); ashish.a.deshmukh@uth.tmc.edu (A.A.D.); 3 Department of Pediatrics, Baylor College of Medicine, Houston, TX 77030, USA; 4 Dan L Duncan Comprehensive Cancer Center, Baylor College of Medicine, Houston, TX 77054, USA

**Keywords:** HPV, human papillomavirus, HPV vaccine, health disparities, racial/ethnic minority

## Abstract

Background: Racial/ethnic minorities generally have a lower knowledge of human papillomavirus (HPV) and the HPV vaccine than non-Hispanic Whites. They are also less likely to have a regular healthcare provider (HCP). Given the role of HCPs in disseminating health information, we evaluated whether racial/ethnic disparities in HPV knowledge are moderated by regular HCP status. Methods: Data from the Health Information National Trends Survey Five (HINTS 5) Cycles One and Two (2017–2018) were analyzed. HPV and HPV vaccine knowledge were compared by regular HCP status across race/ethnicities. Independent partially-adjusted multivariable logistic regression models were used to assess the association between race/ethnicity and knowledge after controlling for sociodemographic characteristics. The resulting adjusted odds ratios were compared to those from fully-adjusted models that included HCP status. Results: After adjusting for regular HCP status, differences in knowledge persisted between racial/ethnic groups. Compared to Whites, Hispanics and Other race/ethnicities had significantly lower odds of having heard of HPV. Blacks, Hispanics, and Other race/ethnicities had significantly lower odds of having heard of the HPV vaccine. Conclusion: Racial/ethnic minorities had significantly lower levels of knowledge despite HCP status. These data suggest the need to address disparities in health information and strengthen provider–patient communication regarding HPV and the HPV vaccine.

## 1. Introduction

Human papillomavirus (HPV) is the most common sexually transmitted infection in the United States (U.S.), with nearly 80 million Americans currently infected and 14 million new infections expected each year [1]. HPV primarily causes six cancers (cervix, anus, vagina, vulva, penis, and oropharynges) [1,2]. Over 45,000 new cases of HPV-associated cancer are reported each year in the U.S. [3]. In an era of overall declining rates of cancers, the incidence of certain HPV-associated cancers (specifically oropharyngeal, anal, and vulvar cancers) is on the rise [4,5]. The HPV vaccine is routinely recommended for boys and girls ages 11–12 years, with catch-up vaccination through age 26 years [1]. Shared patient–provider decision-making about the vaccine is recommended for adults aged 27–45 years [6]. Despite these recommendations, HPV vaccination rates in the U.S. are low, with only 56.8% of all adolescents receiving recommended doses in 2019 [7].

Racial/ethnic minority populations, particularly non-Hispanic Blacks and Hispanics, are disproportionately impacted by HPV and certain HPV-associated cancers. For example, non-Hispanic Black women have a 25% higher incidence of cervical cancer compared to non-Hispanic White women and are almost twice as likely to die from cervical cancer [8]. Among Hispanic women, cervical cancer incidence and mortality are 53% and 41% higher than among non-Hispanic White women [9,10]. The incidence of HPV-associated anal cancer is also rising rapidly among recent birth cohorts of Black men [4]. Given the risk of elevated HPV-associated cancer-elevated mortality, prevention through the HPV vaccine (and regular screening, in the case of cervical cancer) is particularly important for these racial/ethnic minorities [11].

Vaccine and other preventive care uptake is a complex, multilayered phenomenon influenced by factors at the personal, interpersonal, structural, and societal level [12]. Knowledge of HPV, HPV vaccination, and its association with cancers are among the personal cognitive factors associated with HPV vaccine uptake and cervical cancer screening [13]. The literature suggests that racial/ethnic minorities are much less knowledgeable and aware of HPV and the HPV vaccine compared to non-Hispanic Whites [14,15,16]. However, racial/ethnic minorities generally have increased willingness to vaccinate when they receive accurate information from trusted sources, including trusted healthcare providers (HCPs) [17].

HCPs play an important role in the acceptance and uptake of the HPV vaccine and cervical cancer screening [18,19]. A high-quality recommendation by an HCP increases HPV vaccination series initiation and completion by 3- and 9-fold, respectively [20,21] and is known to be one of the strongest predictors of HPV vaccine uptake [22,23]. Recommendations by HCPs are particularly important to increase HPV vaccine uptake in minority populations [23]. Receipt of a recommendation increases the likelihood of HPV vaccination in Hispanics and non-Hispanic Blacks by 40% and 27%, respectively, compared to non-Hispanic Whites [24]. HCPs can also significantly influence women’s cervical cancer screening participation decisions [25,26,27].

Given the role of HCPs in recommending HPV prevention services, we hypothesized that having a regular healthcare provider may attenuate racial/ethnic disparities in knowledge of HPV and the HPV vaccine. Here we examine racial/ethnic differences in knowledge of HPV and the HPV vaccine and assess how they are moderated by having a regular healthcare provider.

## 2. Materials and Methods

### 2.1. Data Source

Cross-sectional data were obtained from the fifth iteration of the National Cancer Institute’s (NCI) Health Information National Trends Survey (HINTS 5), Cycles 1 (2017) and 2 (2018). HINTS is a nationally representative random-digit dial telephone survey of non-institutionalized U.S. adults to monitor data on health-related behaviors, knowledge, and attitudes [28,29]. Data collection ran from January to May 2017 for Cycle 1 (response rate–32.4%) and from January–May 2018 for Cycle 2 (response rate = 32.9%) [28,29]. The data collection methodology for HINTS is described in detail elsewhere [28,29]. The HINTS 5 was granted exempted status by the Internal Review Board (IRB) of the NCI’s Office of Human Subjects Research and by the IRB of the organization that administers the survey, Westat. The current analysis was exempted from review by the IRB of the Baylor College of Medicine.

### 2.2. Measures

Knowledge of HPV and the HPV vaccine were assessed based on the items, “*Have you heard of HPV?*” and “*Have you ever heard of the cervical cancer vaccine or HPV shot?*” Having a regular healthcare provider was assessed through the item, “*Is there a doctor/nurse/health professional that you see most often?*” Sociodemographic variables were race/ethnicity (categorized as non-Hispanic white, non-Hispanic Black, Hispanic, or Other), gender, age, education, and household income. Census region (categorized as Northeast, Midwest, South, and West) and geographic area (categorized as non-metro rural and metro urban) were also available in the HINTS data files. Discussion of the HPV vaccine with a healthcare provider for the ad hoc analysis was assessed through the item “*Has a doctor ever talked with you about the HPV shot?*” This item was only asked to those individuals who reported having an immediate family member between the ages of 9 and 27 years old.

### 2.3. Statistical Analysis

Descriptive statistics were used to describe within-group frequencies and between-group comparisons. Wald chi-square tests and adjusted *p*-values were used for comparisons by race/ethnicity, both overall and stratified by HCP status. Independent, partially-adjusted multivariable logistic regression models were used to examine the association between HPV and HPV vaccine knowledge and awareness in each racial/ethnic group after adjusting for sociodemographic characteristics. A second, fully-adjusted model for each outcome, which included sociodemographic characteristics and regular HCP status, was used to determine whether the latter had an impact on the association. All statistical tests were considered significant if the *p*-value was less than 0.05. An ad hoc analysis was conducted to examine the receipt of a provider recommendation among individuals with a regular HCP who were themselves or who had a family member who was age-eligible for the HPV vaccine (i.e., age 9–26 years). To account for the complex survey design and provide representative estimates of the U.S. population, data from Cycles 1 and 2 were weighted based on the analysis recommendations given by the HINTS investigators [30,31]. This weighting employed household-level base weights, adjusting for household nonresponse, person-level initial weights, and calibrations of person-level weights to population counts. Further, the jackknife variance estimation technique was used to calculate replicate weights [28,29]. Datasets for Cycles 1 and 2 were then merged via the analytic recommendations for merging HINTS iterations given by the HINTS guidance [30,31]. All data analyses were conducted using SAS version 9.4 [32].

## 3. Results

Of the 6552 respondents, 4093 were non-Hispanic White, 1057 were non-Hispanic Black, 778 were Hispanic, and 624 were of Other race/ethnicity. Table 1 compares sociodemographic characteristics by race/ethnicity.

**Figure 1:**
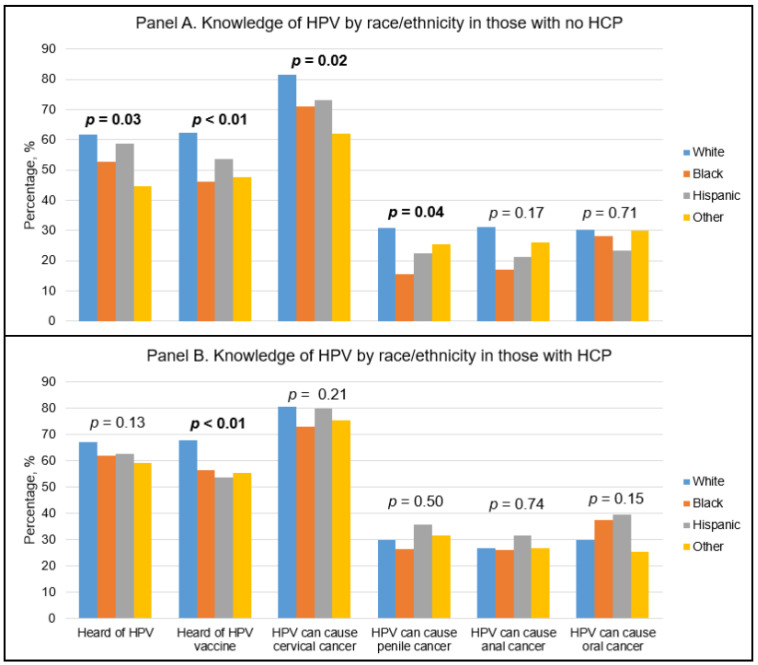
HPV awareness by racial/ethnic group stratified on HCP status; *p*-values denote differences between racial/ethnic groups (White, Black, Hispanic, and Other).

Figure 1 illustrates HPV and HPV vaccine knowledge for race/ethnicities without and with a regular HCP. In those without a regular HCP, there were significant differences between racial/ethnic groups in HPV knowledge (Figure 1A). Minority groups, including non-Hispanic Blacks, Hispanics, and Other race/ethnicities had significantly lower knowledge of HPV, the HPV vaccine, and HPV’s association with cervical cancer and penile cancer. Among those with a regular HCP, knowledge of the HPV vaccine was lowest among Hispanics, followed by Other race/ethnicities, non-Hispanic Blacks, and non-Hispanic Whites (Figure 1B).

**Figure 2:**
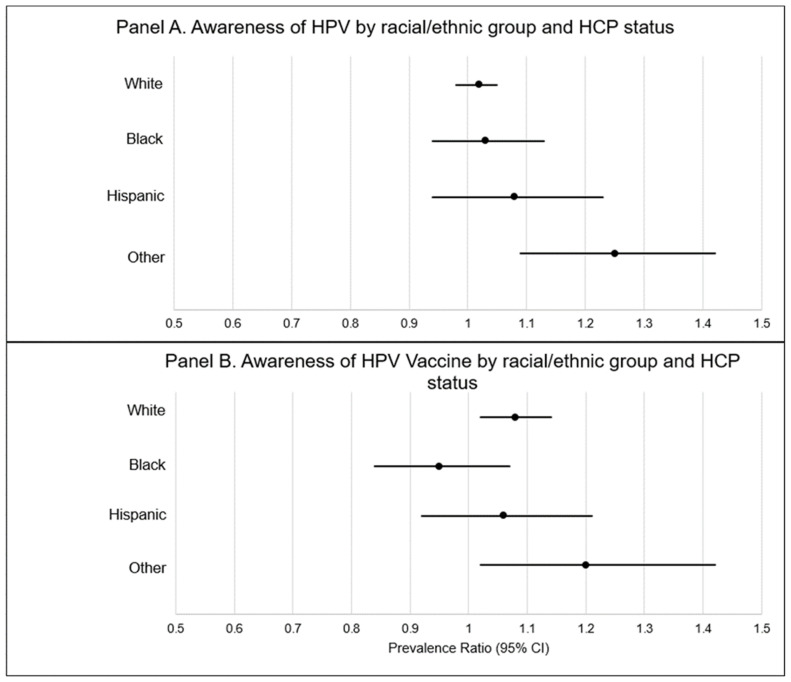
Awareness of HPV and the HPV vaccine within racial/ethnic groups by HCP status. Solid black dot denotes prevalence ratio. Black line denotes 95% confidence interval.

Figure 2 describes differences in knowledge of HPV and the HPV vaccine within racial/ethnic groups by regular HCP status. In this figure, prevalence ratios (PRs) compare prevalent knowledge of HPV and the HPV vaccine by racial/ethnic group among those without a regular healthcare provider (panel A) compared to those with a regular healthcare provider (panel B). Knowledge of HPV was not significantly impacted by HCP status in non-Hispanic Whites, non-Hispanic Blacks, or Hispanics. However, individuals of Other race/ethnicities with an HCP had a higher prevalence of having heard of HPV compared to those without an HCP (PR = 1.25 [95% CI: 1.09–1.42]). Knowledge of the HPV vaccine was not significantly impacted by HCP status among non-Hispanic Blacks or Hispanics. Non-Hispanic Whites and other race/ethnicities with an HCP had a higher prevalence of having heard of the HPV vaccine (PR = 1.08 [95% CI: 1.02–1.14] and PR = 1.20 [95% CI: 1.01–1.42], respectively) when compared to those without an HCP.

The results of the partially-adjusted multivariate logistic regression models (Model 1) comparing odds of HPV and HPV vaccine knowledge while adjusting for sex, age, education, income, census region, and geographic area, are presented in Table 2. The odds for knowledge of HPV, HPV vaccine, and the link between HPV and cervical and oral cancers were significantly lower among racial/ethnic minorities compared to non-Hispanic Whites. These differences persisted in a fully-adjusted model (Model 2) that accounted for HCP status. When adjusted for having a regular HCP, Hispanics had 0.69 (95% CI: 0.50–0.95) times the odds of having heard of HPV and 0.57 (95% CI: 0.39–0.82) times the odds of having heard of the HPV vaccine when compared to non-Hispanic Whites. Further, non-Hispanic Blacks had 0.61 (95% CI: 0.46–0.81) times the odds of having heard of the HPV vaccine when compared to non-Hispanic Whites. Additionally, other races/ethnicities had 0.39 (95% CI: 0.27–0.57) times the odds of having heard of HPV and 0.41 (95% CI: 0.26–0.66) times the odds of having heard of the HPV vaccine when compared to non-Hispanic Whites.

Racial/ethnic differences in knowledge of HPV-associated cancers were mostly non-significant except for cervical and oral cancer (Table 2). In the partially-adjusted multivariable regression model, knowledge of the association between HPV and oral cancer was significantly lower among individuals of Other race/ethnicity compared to non-Hispanic Whites (OR = 0.55 [95% CI: 0.31–0.995] and OR = 0.51 [95% CI: 0.28–0.91], respectively). The adjusted odds ratio remained similar after further adjustment for HCP status. For oral cancer, in both the partially- and fully-adjusted models, knowledge of the association between HPV and oral cancer was significantly lower among non-Hispanic Blacks compared to non-Hispanic Whites (OR = 0.68 [95% CI: 0.47–0.99] and OR = 0.67 [95% CI: 0.46–0.97], respectively). The unadjusted regression model is shown in Table S1.

In ad hoc analyses examining receipt of a provider recommendation among individuals with a regular HCP who were themselves or who had a family member who was age-eligible for the HPV vaccine, individuals of Other race/ethnicity had lower odds (OR = 0.44 (0.20–0.95)) of receiving a provider recommendation compared to non-Hispanic Whites (Table S2). Odds of a provider recommendation were not statistically significantly different for non-Hispanic Blacks and Hispanics, compared to non-Hispanic Whites.

## 4. Discussion

The findings of this nationally representative analysis show that racial/ethnic minority groups in the U.S. have significantly lower knowledge of HPV and the HPV vaccine compared to non-Hispanic Whites. Furthermore, certain minority groups also had significantly lower knowledge of the causal link between HPV and cervical cancer (Other races) or oral cancer (non-Hispanic Blacks) compared to non-Hispanic Whites. Given that a regular healthcare provider is more likely to recommend preventative services (such as the HPV vaccine and cervical cancer screening) and counsel patients during preventative service encounters [18,19], we hypothesized that racial/ethnic disparities in HPV knowledge would be attenuated among individuals who reported having a regular HCP. Although our data suggest having a regular HCP slightly improves HPV knowledge across racial/ethnic groups, differences in knowledge persist.

We hypothesize that the null effect of regular HCP status on the association between race/ethnicity and knowledge of HPV and the HPV vaccine is due to the general low prevalence of HPV and HPV vaccine discussion between HCPs and patients. While a strong recommendation by a provider is a prominent predictor of HPV vaccine uptake [21], many HCPs may not engage in discussing or recommending the vaccine to their patients [33]. In our ad hoc analysis, less than 39% of participants who were themselves age-eligible for the HPV vaccine, or who had a family member who was age-eligible, reported discussing the vaccine with their HCP. This finding is consistent with a recent NIS study which reported that nearly one-half of HPV unvaccinated adolescents in 2018 did not receive a vaccine recommendation from their healthcare provider [34]. There are many reasons for the low prevalence of HPV vaccine discussions during HCP visits. Providers may themselves have a low knowledge of HPV and the HPV vaccine [35,36,37,38] and/or have low confidence and self-efficacy for talking with patients and parents about the HPV vaccine [38,39,40,41]. Providers often miss opportunities to recommend the HPV vaccine due to time constraints, as well as incomplete vaccine records and other challenges with electronic health records systems [41,42]. Finally, some providers may perceive that their patients are vaccine-hesitant [43] or underestimate the importance of the HPV vaccine to parents [44,45,46,47].

In our ad hoc analysis of individuals with a regular healthcare provider who were themselves age-eligible for the HPV vaccine or had an age-eligible family member, we found that the prevalence of HPV vaccine discussion with a provider was not significantly different for non-Hispanic Blacks and Hispanics compared to non-Hispanic Whites. Our ad hoc analysis had a relatively small sample size and was underpowered to detect meaningful differences. However, prior studies have reported significant racial/ethnic disparities in provider HPV vaccine recommendations [48]. While further data are needed to evaluate this finding, it is possible that the null effect of HPC status on HPV vaccine knowledge among race/ethnic minorities was not discerned due to the disproportionately low prevalence of HPV vaccine discussions by HCPs. This underlying issue of the low prevalence of HPV vaccine discussions among minorities could have rendered our sample size insufficient for our ad hoc analysis.

Our findings suggest that having access to a regular HCP does not mitigate disparities in knowledge of HPV or the HPV vaccine among racial/ethnic minority populations. This informs future opportunities to explore other options that may contribute to disparate knowledge such as the communication strategy used for patient–provider discussions regarding vaccination [49] especially with racial/ethnic minority populations [49]. As HCPs have been shown to play a significant role in individuals’ willingness and intent to vaccinate, it is important that HCPs are equipped with evidence-based communication tools, such as presumptive recommendations, to discuss the HPV vaccine with their patients [49,50].

There are limitations to this research that should be considered when interpreting these results. As with all survey data, there is room for recall bias, particularly when reporting provider HPV vaccine recommendations. However, we are unaware of any data indicating that recall bias is differential across race/ethnicities. Our ad hoc analysis had a small sample size resulting in its low power to detect differences in HPV vaccine recommendation by regular HCP. Finally, while HCP status is a common marker of healthcare access and utilization [51], the variable does not capture important characteristics, including the type of HCP and the frequency of healthcare encounters. The strengths of our study include our use of a nationally representative sample. Additionally, our statistical models were comprehensive and included all relevant sociodemographic variables.

## 5. Conclusions

In conclusion, having a regular HCP does not seem to attenuate the significant racial/ethnic disparities in HPV and HPV vaccine knowledge. Further research is needed to understand factors contributing to HPV knowledge differences and the subsequent development of targeted interventions to mitigate racial/ethnic disparities in knowledge and awareness of HPV and the HPV vaccine.

## Figures and Tables

**Table 1:** Weighted respondent characteristics by racial/ethnic group, HINTS 5 Cycle 1 and 2, 2017–2018 (*n* = 6552).

	White (*n* = 4093)	Black (*n* = 1057)	Hispanic (*n* = 778)	Other (*n* = 624)	Adjusted *p*-Value ^a^
	*n* (column%)	*n* (column%)	*n* (column%)	*n* (column%)	
Gender					0.55
Male	1724 (49.26)	321 (45.93)	316 (49.89)	256 (51.67)	
Female	2319 (50.74)	707 (54.07)	456 (50.11)	361 (48.33)	
Age, years					**<0.0001**
18–34	427 (20.91)	91 (15.11)	143 (30.59)	104 (33.97)	
35–49	692 (24.82)	219 (33.09)	215 (34.96)	161 (29.87)	
50–64	1337 (30.84)	391 (38.00)	233 (25.48)	186 (24.32)	
65–74	939 (13.31)	224 (9.24)	114 (6.01)	106 (6.62)	
75+	639 (10.13)	99 (4.55)	61 (2.97)	57 (5.22)	
Education					**<0.0001**
Less than HS	167 (5.83)	136 (16.39)	127 (16.14)	52 (9.43)	
HS graduate or equivalent	737 (21.07)	231 (27.56)	167 (28.04)	80 (17.41)	
Some college	1211 (40.66)	335 (30.59)	210 (26.54)	184 (28.86)	
College graduate or post graduate	1952 (32.44)	344 (25.46)	265 (29.28)	301 (44.31)	
Household Income					**<0.0001**
<$20,000	488 (14.00)	337 (34.41)	167 (17.16)	120 (19.22)	
$20,000–$34,999	471 (11.57)	175 (13.59)	121 (15.06)	65 (8.92)	
$35,000–$49,999	461 (13.19)	129 (14.46)	92 (17.12)	91 (16.26)	
$50,000–$74,999	710 (18.47)	143 (14.71)	114 (18.44)	112 (21.94)	
$75,000+	1571 (42.78)	170 (22.84)	215 (32.22)	195 (33.66)	
Census Region					**<0.0001**
Northeast	680 (17.90)	136 (15.73)	109 (16.42)	101 (20.68)	
Midwest	916 (26.20)	187 (16.44)	47 (8.18)	63 (10.82)	
South	1608 (35.04)	649 (59.67)	322 (32.77)	207 (33.38)	
West	889 (20.85)	85 (8.16)	300 (42.63)	253 (35.12)	
Geographic Area					**<0.0001**
Rural	689 (17.42)	96 (8.27)	55 (5.29)	56 (9.76)	
Urban	3404 (82.58)	961 (91.73)	723 (94.71)	568 (90.24)	
Regular Provider					**<0.0001**
Yes	3180 (72.71)	667 (56.18)	424 (47.67)	373 (53.13)	
No	871 (27.29)	375 (43.82)	348 (52.33)	238 (46.87)	

Boldface indicates statistical significance (*p* < 0.05); ^a^ *p*-values denote differences between racial/ethnic groups (White, Black, Hispanic, Other).

**Table 2:** Weighted multivariate logistic regression analyses examining HPV knowledge and awareness by racial/ethnic group.

	Race/Ethnicity	Model 1 ^a^	Model 2 ^b^
		Adjusted OR ^c^ (95% CI) ^d^	Adjusted OR (95% CI)
Heard of HPV	White	1.00	1.00
	Black	0.747 (0.507–1.102)	0.77 (0.51–1.15)
	Hispanic	**0.664 (0.493–0.893)**	**0.69 (0.50–0.95)**
	Other	**0.391 (0.261–0.584)**	**0.39 (0.27–0.57)**
Heard of HPV vaccine	White	1.00	1.00
	Black	**0.607 (0.460–0.801)**	**0.61 (0.46–0.81)**
	Hispanic	**0.550 (0.381–0.795)**	**0.57 (0.39–0.82)**
	Other	**0.419 (0.271–0.647)**	**0.41 (0.26–0.66)**
HPV can cause cervical cancer	White	1.00	1.00
	Black	0.716 (0.489–1.047)	0.72 (0.49–1.06)
	Hispanic	0.747 (0.468–1.192)	0.78 (0.49–1.24)
	Other	**0.553 (0.308–0.995)**	**0.51 (0.28–0.91)**
HPV can cause oral cancer	White	1.00	1.00
	Black	**0.682 (0.471–0.987)**	**0.67 (0.46–0.97)**
	Hispanic	1.105 (0.722–1.691)	1.15 (0.73–1.79)
	Other	1.109 (0.682–1.806)	1.14 (0.69–1.88)
HPV can cause anal cancer	White	1.00	1.00
	Black	0.708 (0.449–1.118)	0.69 (0.43–1.1)
	Hispanic	0.960 (0.621–1.484)	0.98 (0.62–1.5)
	Other	1.000 (0.665–1.506)	1.02 (0.67–1.55)
HPV can cause penile cancer	White	1.00	1.00
	Black	1.194 (0.777–1.836)	1.20 (0.78–1.85)
	Hispanic	1.230 (0.862–1.754)	1.30 (0.92–1.85)
	Other	0.928 (0.646–1.333)	0.97 (0.67–1.40)

^a^ Model 1 has been adjusted for selected demographic factors (sex, age, education, household income, census region, geographic area); ^b^ Model 2 has been adjusted for selected demographic factors listed in Model 2 and having a regular healthcare provider; ^c^ OR = odds ratio; ^d^ CI = confidence interval. Boldface indicates statistical significance (*p* < 0.05).

## Data Availability

Publicly available datasets were analyzed in this study. This data can be found here: https://hints.cancer.gov/data/default.aspx (accessed on 20 July 2021).

## Supplementary Materials

The following are available online at https://www.mdpi.com/article/10.3390/vaccines9070802/s1. The first supplementary table is titled Table S1: Weighted Univariable Logistic Regression Analyses Examining HPV Knowledge and Awareness by Racial/Ethnic Group, Table S1 presents the unadjusted logistic regression modeling supplementary to the main analyses presented in Table 2. The second supplementary table is titled Table S2: Ad Hoc Analysis: Discussion of HPV Vaccination with Healthcare Provider in Those Who Had a Regular Healthcare Provider by Race/Ethnicity, Table S2 presents the ad hoc analyses that examined the receipt of a provider recommendation among individuals with a regular healthcare provider who were themselves or who had a family member who was age-eligible for the HPV vaccine.
