# Supplementary material for: Regular Healthcare Provider Status Does Not Moderate Racial/Ethnic Differences in Human Papillomavirus (HPV) and HPV Vaccine Knowledge
Source: Vaccines (Basel). 2021 Jul 20;9(7):802. doi: 10.3390/vaccines9070802 (PMC8310170; doi:10.3390/vaccines9070802)
Supplement: Supplementary file 1 [file vaccines-09-00802-s001.zip › vaccines-1271839-supplementary.pdf]

Supplementary Tables

Table S1. Weighted Univariable Logistic Regression Analyses Examining HPV Knowledge and Awareness by Racial/Ethnic Group

	Race/Ethnicity	Model 1<br>Unadjusted OR[a] (95% CI)[b]
Heard of HPV	White	1.00
	Black	**0.721 (0.544 - 0.954)**
	Hispanic	0.841 (0.682 - 1.036)
	Other	**0.587 (0.447 - 0.771)**
Heard of HPV vaccine	White	1.00
	Black	**0.547 (0.422 - 0.708)**
	Hispanic	**0.582 (0.458 - 0.741)**
	Other	**0.548 (0.400 - 0.750)**
HPV can cause cervical cancer	White	1.00
	Black	**0.608 (0.428 - 0.863)**
	Hispanic	0.745 (0.513 - 1.082)
	Other	**0.573 (0.356 - 0.923)**
HPV can cause oral cancer	White	1.00
	Black	**0.674 (0.483 - 0.941)**
	Hispanic	0.926 (0.620 - 1.382)
	Other	0.924 (0.589 - 1.450)
HPV can cause anal cancer	White	1.00
	Black	0.763 (0.526 - 1.106)
	Hispanic	0.898 (0.599 - 1.345)
	Other	0.907 (0.590 - 1.395)
HPV can cause penile cancer	White	1.00
	Black	1.191 (0.861 - 1.648)
	Hispanic	1.037 (0.732 - 1.468)
	Other	0.851 (0.592 - 1.222)

a. OR = odds ratio
b. CI = confidence interval
Boldface indicates statistical significance ($p<0.05$)

Table S2. Ad-Hoc Analysis: Discussion of HPV Vaccination with Healthcare Provider in Those Who Had A Regular Healthcare Provider by Race/Ethnicity[a]		
	%	Age-Adjusted OR (95% CI)
White, N=357	38.0	1.00
Black, N=117	38.5	0.98 (0.53 - 1.83)
Hispanic, N=82	38.6	0.98 (0.42 - 2.25)
Other, N=66	21.8	**0.44 (0.20 - 0.95)**

a. Participants only answered this question is they reported having family member in their household between ages 9-27 years

Boldface indicates statistical significance ($p<0.05$)
